# Salmonella Prostatic Abscess in an Immunocompetent Patient

**DOI:** 10.7759/cureus.9122

**Published:** 2020-07-10

**Authors:** Christina Lin, Satya Patel

**Affiliations:** 1 Internal Medicine, University of California Los Angeles, Los Angeles, USA

**Keywords:** salmonella, fever, bacteremia, prostatic abscess, travel

## Abstract

Patients returning from travel to a foreign country often present with atypical infections that can present as diagnostic challenges. Although rarely seen in the United States, Salmonella infections are commonly seen in Sub-Saharan Africa. The common clinical manifestations of Salmonella infection include fever and diarrhea; however, about 5% of cases of non-typhi Salmonella progress to bacteremia. Here, we present a case of a unique presentation of Salmonella infection manifesting as a prostatic abscess in an immunocompetent patient.

## Introduction

Non-typhoidal salmonellosis is a common cause of diarrhea worldwide though occurs most frequently in Sub-Saharan Africa, where 79% of all cases globally are found [1]. Other endemic regions include South Asia, Latin America, and the Middle East. Risk factors for disease include an immunocompromised state, malaria, hemoglobinopathies (including sickle cell trait), chronic liver disease, and chronic granulomatous disease. Non-typhoidal Salmonella are associated with ingestion of poultry, milk products, eggs, fresh produce, and contact with animals. Common features of infection include fever, abdominal pain, and diarrhea. Most infections are self-limited; however, up to 5% of cases result in bacteremia and extraintestinal manifestations. Risk factors for more invasive disease include infants (<12 months) or older age (>50 years), HIV, or any other immunocompromising condition. Invasive disease is seen most commonly in non-typhoidal Salmonella serovars that are endemic to Sub-Saharan Africa [2-4].

## Case presentation

A 50-year-old immunocompetent male with past medical history significant for childhood malaria presented with fevers and myalgias following a trip to Ghana one week prior. He initially denied any focal infectious symptoms, including cough, chest pain, shortness of breath, dysuria, nausea, abdominal pain, or diarrhea. During the trip, he stayed in a rural village with indoor plumbing, ate home-cooked food, drank bottled water, and was not aware of any sick contacts.

His presenting vital signs were as follows: temperature 102˚F, heart rate 133 bpm, blood pressure 127/88 mmHg, and oxygen saturation 98% on ambient air. On physical exam, he appeared fatigued with dry mucous membranes, was tachycardic with a regular rhythm, but otherwise had no significant findings. His laboratory testing showed a white blood cell count of 12,000/µL with 86.1% neutrophils and 70 bands/µL. Initial blood cultures, urinalysis, and chest X-ray were unremarkable. Due to his recent travel and concern for infection, a broad infectious work-up was done. Nasal influenza/respiratory syncytial viral swab, HIV-1/HIV-2, dengue IgM, Zika IgM, and blood smears for malaria were negative. Given his persistent high fevers and recent travel to an endemic area for malaria, he was empirically treated for malaria with atovaquone/proguanil without significant improvement in his symptoms. On hospital day 2, the patient had a temperature to 101.6˚F and developed dysuria and gross hematuria. A repeat urinalysis demonstrated pyuria and 2+ blood, and he was empirically started on ceftriaxone.

**Figure 1:**
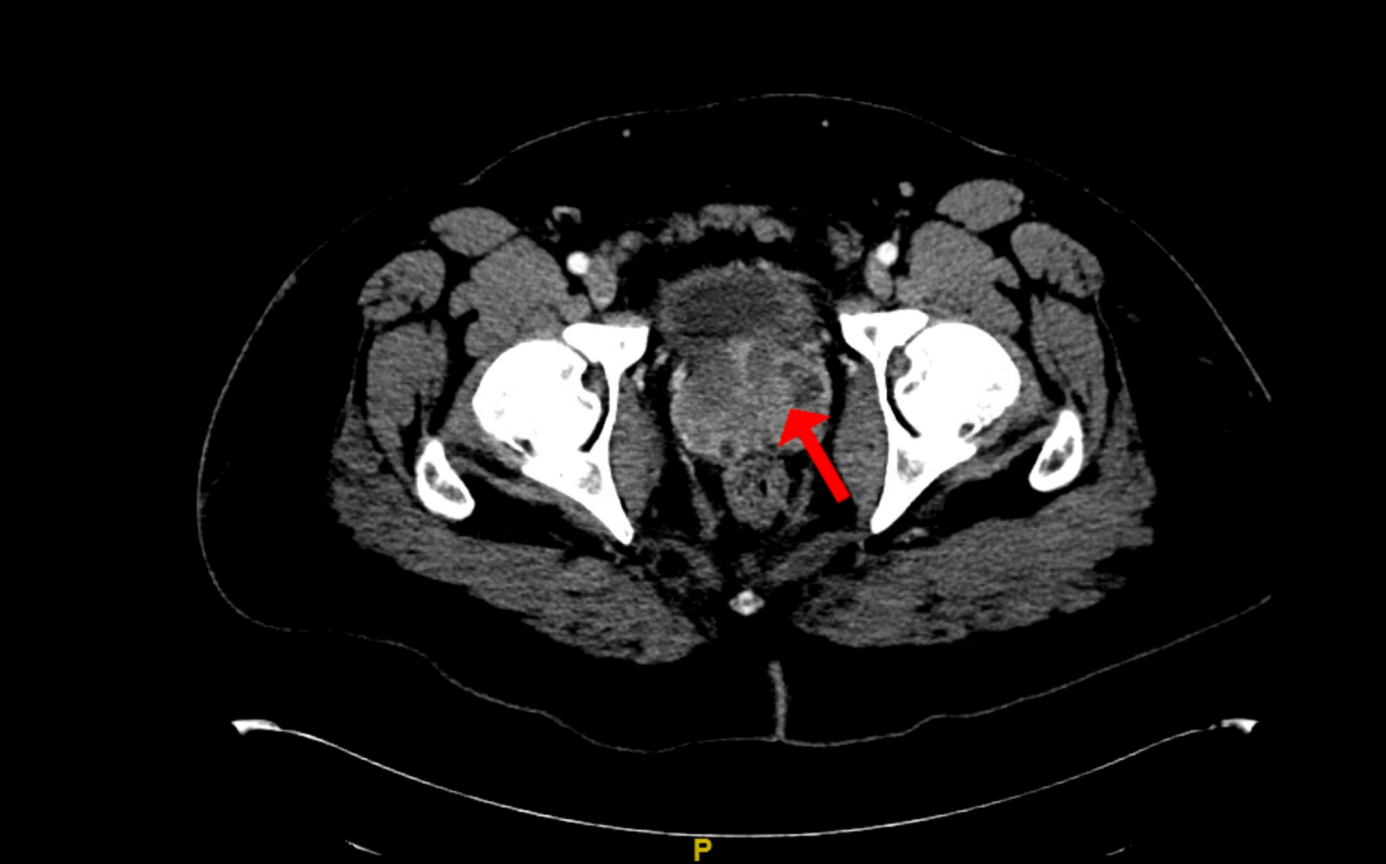
CT of the abdomen/pelvis with contrast demonstrating a moderately enlarged, heterogeneous appearing prostate gland with multiple hypoattenuating foci within it and surrounding inflammatory change concerning for multifocal prostatic abscesses.

His urine culture later revealed ceftriaxone-sensitive Salmonella non-typhi Group B serotype. The patient continued to have fevers and on hospital day 4, the patient developed non-bloody diarrhea. Stool bacterial testing demonstrated the same strain of Salmonella. Blood cultures continued to be negative. Due to his persistent fevers, a CT scan of his abdomen and pelvis with intravenous contrast was obtained and showed multifocal prostatic abscesses (Figure 1). The patient underwent transgluteal drainage of his prostatic abscesses and the resultant cultures grew the same pathogen. His fever curve subsequently improved, and he was discharged on an extended course of trimethoprim-sulfamethoxazole.

## Discussion

The most common manifestation of Salmonella infection is gastroenteritis. The pathogenesis of infection involves ingestion of the bacteria into the gastrointestinal tract, whereby the microorganism must survive in the acidic environment of the stomach, and eventual adherence and invasion of the M cells within the Peyer patches of the colon. Alterations of the gastrointestinal tract, such as acid-reducing medications or antibiotic use, can increase the risk of progression from enteric to systemic salmonellosis [5]. Bacteremia and disseminated infection is estimated to occur in about 5% of cases, most commonly with the higher virulence strains of non-typhi Salmonella [6]. Salmonella bacteremia can result in infection at almost any site, the most common sites of dissemination being urinary, lung, bones, and central nervous system. A study in Malaysia found that immunocompromised patients with Salmonella infection not only had a higher mortality but also were more likely to present with primary bacteremia compared to their immunocompetent counterparts [7]. Thus, a patient with disseminated salmonellosis should be worked up for any underlying immunocompromising condition. Uncomplicated disease can be treated with supportive care without antibiotics; however, those with extraintestinal complications require a prolonged antibiotic course and often surgical interventions.

This case represents a unique presentation of Salmonella infection manifesting as a prostatic abscess. Prostatic abscesses due to Salmonella are exceedingly rare and have previously only been described in immunocompromised patients, which was not the case in our patient [8]. Most cases of extraintestinal salmonellosis are a result of hematological spread from gut translocation [9,10]. Although it is unclear whether the primary source of infection in this case was gut translocation or ascending urinary tract infection, spread between the sites was most likely hematologic as bacteremia is a known complication of Salmonella infections. This raises a diagnostic dilemma as to how to interpret the negative blood cultures. The sensitivity for non-typhoid Salmonella on blood cultures is unknown, although it is notable that the sensitivity of typhoid Salmonella on blood cultures is only 50%-70% [11]. It is therefore of high utility to obtain a detailed clinical exam to assess for evidence of disseminated salmonellosis even in the presence of negative blood cultures.

## Conclusions

A patient traveling from endemic areas of Salmonella infection such as Africa should raise clinical suspicion for salmonellosis. Mortality and morbidity from disseminated Salmonella infections are high. Clinicians should be aware of the extraintestinal complications of disseminated Salmonella infections, which can often serve as diagnostic challenges.
